# *IDH1* mutation detection by droplet digital PCR in glioma

**DOI:** 10.18632/oncotarget.5630

**Published:** 2015-10-14

**Authors:** Jing Wang, Yi-ying Zhao, Jian-feng Li, Cheng-cheng Guo, Fu-rong Chen, Hong-kai Su, Hua-fu Zhao, Ya-kang Long, Jian-yong Shao, Shing-shun Tony To, Zhong-ping Chen

**Affiliations:** ^1^ Department of Neurosurgery/Neuro-oncology, Sun Yat-sen University Cancer Center; State Key Laboratory of Oncology in South China, Collaborative Innovation Center for Cancer Medicine, Guangzhou, China; ^2^ Department of Health Technology and Informatics, The Hong Kong Polytechnic University, Hung Hom, Hong Kong, China; ^3^ Department of Neurosurgery, Affiliated Shantou Hospital of Sun Yat-Sen University, Shantou, China; ^4^ Department of Molecular Diagnosis, Sun Yat-sen University Cancer Center, State Key Laboratory of Oncology in South China, Collaborative Innovation Center for Cancer Medicine, Guangzhou, China

**Keywords:** glioma, isocitrate dehydrogenase (IDH), droplet digital PCR (ddPCR), quantitative real-time PCR (qRT-PCR), sensitivity

## Abstract

Glioma is the most frequent central nervous system tumor in adults. The overall survival of glioma patients is disappointing, mostly due to the poor prognosis of glioblastoma (Grade IV glioma). Isocitrate dehydrogenase (IDH) is a key factor in metabolism and catalyzes the oxidative decarboxylation of isocitrate. Mutations in *IDH* genes are observed in over 70% of low-grade gliomas and some cases of glioblastoma. As the most frequent mutation, *IDH1(R132H)* has been served as a predictive marker of glioma patients. The recently developed droplet digital PCR (ddPCR) technique generates a large amount of nanoliter-sized droplets, each of which carries out a PCR reaction on one template. Therefore, ddPCR provides high precision and absolute quantification of the nucleic acid target, with wide applications for both research and clinical diagnosis. In the current study, we collected 62 glioma tissue samples (Grade II to IV) and detected *IDH1* mutations by Sanger direct sequencing, ddPCR, and quantitative real-time PCR (qRT-PCR). With the results from Sanger direct sequencing as the standard, the characteristics of ddPCR were compared with qRT-PCR. The data indicated that ddPCR was much more sensitive and much easier to interpret than qRT-PCR. Thus, we demonstrated that ddPCR is a reliable and sensitive method for screening the *IDH* mutation. Therefore, ddPCR is able to applied clinically in predicting patient prognosis and selecting effective therapeutic strategies. Our data also supported that the prognosis of Grade II and III glioma was better in patients with an *IDH* mutation than in those without mutation.

## INTRODUCTION

Glioma is a common adult central nervous system tumor. Glioblastoma (GBM), Grade IV glioma, is the most lethal brain tumor (only 12 to 14 months after diagnosis). Treatment response of glioma relies largely on its molecular characteristics. Isocitrate dehydrogenase (IDH) is a key factor in metabolism and catalyzes the oxidative decarboxylation of isocitrate, producing α-ketoglutarate and CO_2_. IDH1 and IDH2 catalyze the same reaction outside the context of the citric acid cycle and use NADP^+^ as a cofactor. Mutations in *IDH* genes (*IDH1* and *IDH2*) are observed in over 70% of low-grade gliomas and some GBM [1, 2]. The most frequent mutation (over 95%) is the G to A mutation on amino acid 132 (CGT > CAT, R132H) at exon 4. The following mutation patterns were also identified: R132C (CGT > TGT), R132L (CGT > CTT), R132S (CGT > AGT), and R132G (CGT > GGT) [3]. Wild-type IDH1 converts isocitrate to α-ketoglutarate (a potential oncometabolite), whereas the mutant IDH1 yields a neomorphic enzymatic function and catalyzes α-ketoglutarate into α-hydroxyglutarate, which is an oncometabolite that is related to genomic hypertension, genetic instability, and malignant transformation [4]. The *IDH1* mutation is one of the most common and earliest genetic alterations in glioma and is an effective diagnostic and predictive marker in glioma patients. Jose et al. [5] investigated The Cancer Genome Atlas (TCGA) data and found that the level of pyruvate carboxylase was higher in human gliomas containing the *IDH1* mutation than in those with wild-type *IDH1*. The fractional flux, which depends on the activity of pyruvate carboxylase, is therefore increased in cells with the *IDH1* mutation. Furthermore, Morteza et al. [6] demonstrated that the mutated IDH1 (R132H) is involved in phosphoethanolamine and glycerophosphocholine and subsequently alters phospholipid metabolism in glioma. Thus, the importance of the *IDH* mutation in the early development of glioma was confirmed. In addition to the important role in glioma, *IDH* mutations were found in myeloid neoplasia, peripheral T-cell lymphoma, chondrosarcoma, chonangiocarcinoma, prostate cancer, and other cancers [7, 8].

Droplet digital PCR (ddPCR) is one of the latest molecular amplification techniques that offers high precision and sensitivity and detects rare alleles, copy number variations and absolute quantification of DNA [9–11]. Its high sensitivity enables the detection of a mutant allele fraction as low as 0.1% [12]. The basic principle of ddPCR relies on the generation of a large number of partitions in the form of nanoliter-sized droplets, each of which carries out a PCR reaction on one template. PCR-positive and PCR-negative droplets are then counted by a specialized droplet reader to provide absolute quantification of target DNA in a digital form and are then analyzed by software. As an “ultra-sensitive detection method”, ddPCR was applied in the pretreatment of EGFR T790M mutations in non-small cell lung cancer patients [13]. Detection of HER2 amplification in gastric cancer by ddPCR was as effective as immunohistochemistry/fluorescence in situ hybridization (IHC/FISH) and may become a standard method for analyzing formalin-fixed paraffin-embedded (FFPE) samples [14]. In glioma, ddPCR successfully measured the *IDH1* mutations in extracellular vesicles and cerebrospinal fluid [15].

In the current study, we collected 62 glioma patient tumor samples and detected *IDH1(R132H)* mutation with ddPCR and qRT-PCR. We evaluated the sensitivity and specificity of ddPCR and qRT-PCR compared with the current standard method, Sanger direct sequencing. We also analyzed the role of *IDH1(R132H)* mutations in the prognosis of low-grade glioma patients.

## RESULTS

### Detection profiles of ddPCR, qRT-PCR, and Sanger sequencing

**Figure 1:**
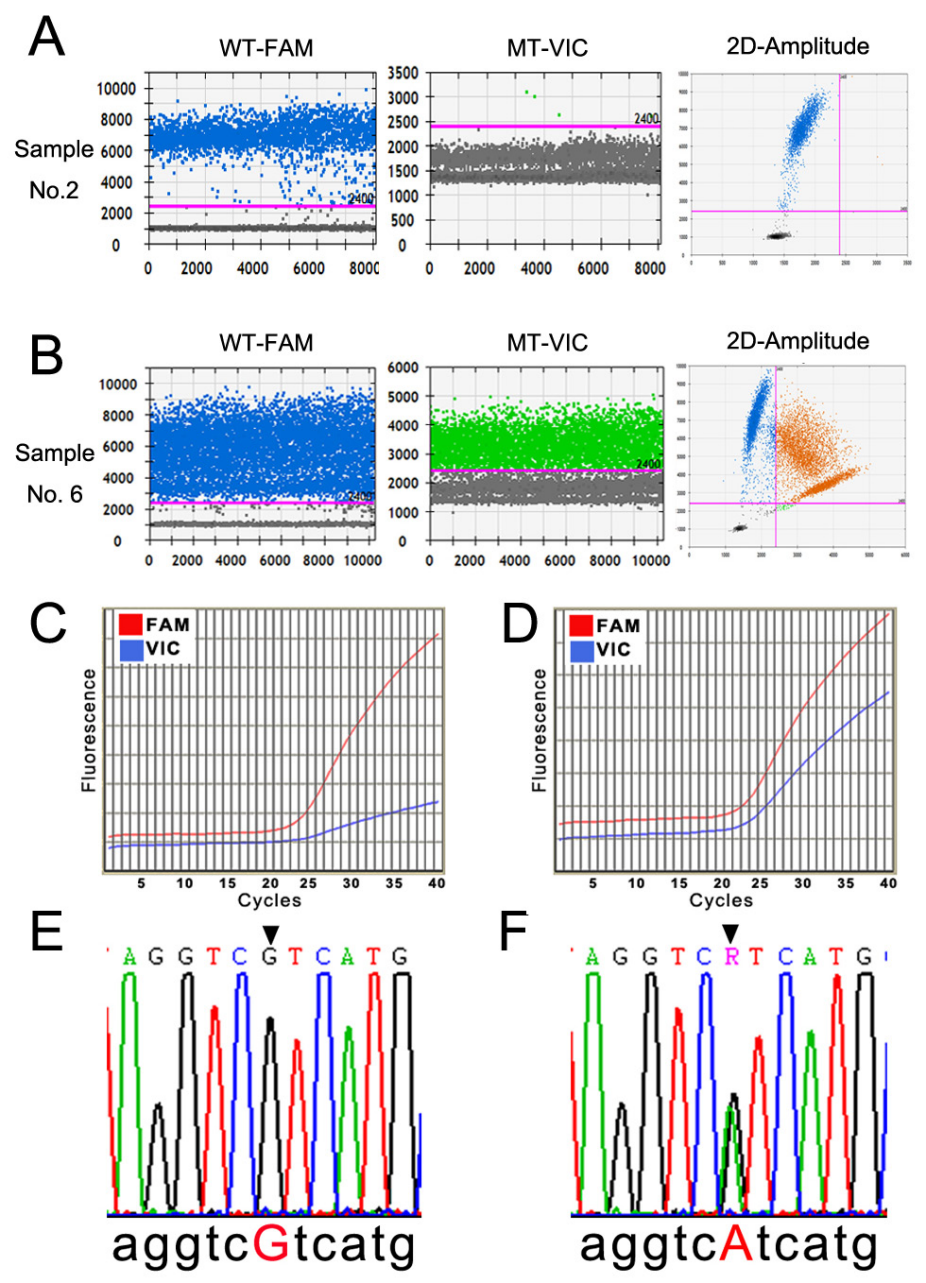
Detection profiles of the *IDH1(R132H)* mutation by ddPCR, qRT-PCR, and Sanger sequencing **A** and **B.** The representative 1-D and 2-D plots of the ddPCR amplification profile of a *IDH1* wild-type and a *IDH1(R132H)* mutant sample. The pink line in the left two rows indicates the threshold, and the orange dots in the upper-right quadrant of the right row indicate the mutant signal. **C** and **D.** The amplification curve of qRT-PCR of a *IDH1* wild-type and a *IDH1(R132H)* mutant sample. The red line denotes the wild-type (WT-FAM) sample, and the blue line denotes the mutant (MT-VIC). **E** and **F.** The results of Sanger sequencing of the wild-type and *IDH1(R132H)* mutant samples (CGT > CAT).

Pilot experiments of ddPCR were performed to establish the assay conditions for *IDH1(R132H)* detection. In 1-D plot, each droplet from a sample was plotted on the graph of fluorescence intensity versus droplet number. The 2-D plot, in which channel 1 fluorescence (wild-type *IDH1*, WT-FAM) was plotted against channel 2 fluorescence (mutant *IDH1*, MT-VIC) for each droplet, indicated that the two targets were amplified at the same time. Channel 1, WT-FAM, was also considered as the assay control to ensure the experimental conditions were sufficient (left panels of Fig. 1A–1B), and the positive dots in channel 2, MT-VIC, indicated the mutation status (middle panels of Figs. 1A–1B). Dots in the upper-right quadrant of the 2-D plot indicated that the sample had the *R132H* mutant (right panels of Fig. 1A–1B). The typical profiles of the wild-type and *R132H* mutants according to ddPCR detection are shown in Fig. 1A and 1B, respectively. There were 21 *R132H* mutants (33.87%) in the 62 samples by ddPCR (Table 1).

**Table 1:** Detection of *IDH1(R132H)* by Sanger sequencing, ddPCR, and qRT-PCR

	Sanger Sequencing	ddPCR	qRT-PCR
Wilde-type	43	41	45
*R132H*	19	21	17
Total	62	62	62

qRT-PCR was performed using the primer pair and probes in the same sample cohort. There were two amplification curves for each target, which was considered amplified if the curve exhibited a sharp increase (blue curve in Fig. 1D) rather than being relatively flat (blue curve in Fig. 1C). The sample had homogenously wild-type *IDH1*, as shown in Fig. 1C, and the sample with heterogeneous wild-type *IDH1* and *R132H* mutations is shown in Fig. 1D. Seventeen mutant samples (27.42%) were detected by qRT-PCR in the 62 samples (Table 1).

As the “golden criteria” in mutation detection, Sanger direct sequencing was applied as a standard in our study [8, 17, 18]. Whole-genomic DNA from glioma patient tumor tissue samples was sequenced after two rounds of PCR reaction following the manufacturer's instructions. The typical data profiles of the wild-type and *R132H* mutant are illustrated in Fig. 1E and 1F. A total of 19 *R132H* mutants (30.65%) were detected among the 62 samples (Table 1).

### Comparison of ddPCR and qRT-PCR

Sensitivity and specificity are two critical characteristics used to evaluate novel detection techniques. Sensitivity, the true-positive rate, indicates the proportion of positives that are correctly identified. Specificity, the true-negative rate, indicates the proportion of negatives that are correctly identified. In our study, the results from ddPCR and qRT-PCR were compared with those derived from Sanger direct sequencing, which is currently considered the gold standard for *IDH1(R132H)* mutation. Sequencing data showed that 19 of the 62 glioma patients (30.65%) had a *IDH1(R132H)* mutation in our cohort, whereas the detection rates of ddPCR and qRT-PCR were 33.87% and 27.42%, respectively. We then calculated the sensitivity and specificity of ddPCR and qRT-PCR based on the direct sequencing results. The sensitivity of ddPCR was very good (100%), whereas it was only 90.48% for qRT-PCR. However, the specificity of ddPCR was 95.56%, compared with the 100% specificity of qRT-PCR (Table 2). Accordingly, the false-positive and false negative rate of ddPCR was 4.44% and 0.00% respectively. The false-positive and false negative rate of qRT-PCR was 0.00% and 9.52% respectively (Table 2).

**Table 2:** Comparison of ddPCR and qRT-PCR

	ddPCR	qRT-PCR
Sensitivity (%)	100	90.48
Specificity (%)	95.56	100
PPV (%)	90.48	100
NPV (%)	100	95.56
False-positive rate (%)	4.44	0
False-negative rate (%)	0	9.52

Furthermore, the positive prediction value (PPV) and negative prediction value (NPV) is another important parameter to evaluate the performance of a new screen test. PPV is the probability of the positive results among true positive detections. NPV is the negative results among the true negative results. The PPV and NPV was 90.48% and 100% for ddPCR, 100% and 95.56% for qRT-PCR respectively (Table 2). Our data therefore suggested that ddPCR performs better in predicting the negative results and fits for screening a large population, whereas qRT-PCR is better predicting the positive results and suits for detection confirmation.

**Figure 2:**
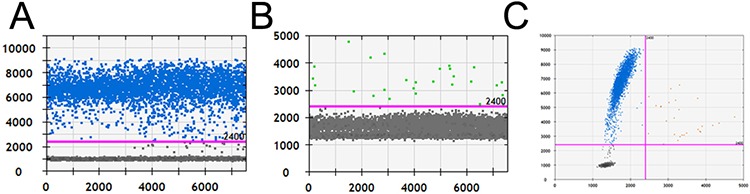
The amplification results of a false-positive sample by ddPCR **A and B.** The 1-D plots of positive channel 1-WT-FAM and false-positive channel 2-MT-VIC. **C.** The 2-D plot of WT-FAM and MT-VIC from a false-positive sample.

Regarding the false-positive rate of ddPCR (4.55%), we chose one of the two samples that yielded inconsistent results with the Sanger direct sequencing results. The wild-type probe signal (channel 1) was adequate according to the 1-D dot plot (Fig. 2A). Although there were few dots above the threshold of channel 2-MT-VIC (Fig. 2B), the positive droplet number was much lower than the true mutant signal as indicated in Fig. 1B. It was not confidently consider the sample to be negative. Accordingly, some dots also appeared in the upper-right quadrant of the 2-D plot (Fig. 2C). Thus, we suggest performing Sanger sequencing to confirm the mutation status if the result of ddPCR is unclear. Although direct sequencing was still considered the standard for mutation detection, a more sensitive method emerged with the improvement in technology.

### Detection limitations of ddPCR and qRT-PCR

**Figure 3:**
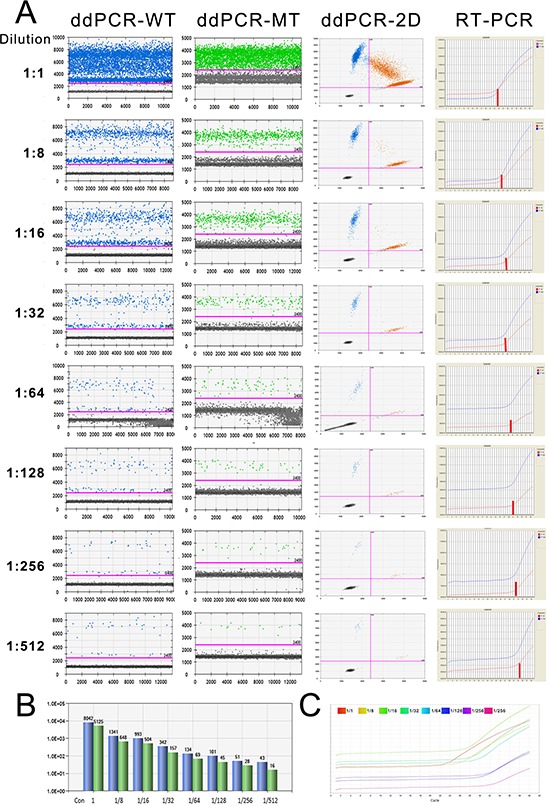
The detection limitations of ddPCR and qRT-PCR **A.** The amplification results of a serially diluted sample derived from ddPCR and qRT-PCR. The number of dots above the threshold decreased following the dilution of the sample (the left three columns show the amplification of WT-FAM and MT-VIC and the 2-D plots with 2 channels). The right column is the amplification curve of qRT-PCR. **B.** The number of wild-type and mutant droplets detected in each diluted template. Blue, wild-type; green, mutant. **C.** The amplification curves of the mutant allele in the serially diluted template according to qRT-PCR.

To determine the difference in the detection sensitivity of a low-concentration template, we performed ddPCR and qRT-PCR with diluted samples (Sample No.19, with *IDH1(R132H)* mutation). In the ddPCR assay, with the decrease in the template concentration, the positive droplet numbers of the wild-type and mutant were decreased in 1-D plots as well as in the upper-right quadrants of the 2-D plot (the left three rows in Fig. 3A). Based on the results of qRT-PCR, we found that the cycle number from which the amplification curve sharply rose increased together with the dilution (right row in Fig. 3A, blue curve indicates wild-type, red curve indicates mutant, short red line indicates the threshold cycle). In particular, we noticed that the mutant droplet number decreased from 5125 to 648, 504, 157, 69, 45, 28, and 16 following dilution at the ratios 1:1, 1:8, 1:16, 1:32, 1:64, 1:128, 1:256, and 1:512, respectively, based on ddPCR results (Fig. 3B). With regard to qRT-PCR, although the cycle in which the amplification curve increased shifted to the right with the decrease in template amount, it was difficult to determine the reduction degree (Fig. 3C). Therefore, our data demonstrated that ddPCR yielded more direct results than qRT-PCR.

### The *IDH1* mutation predicted better prognosis of glioma patients

Direct sequencing data were used for the following correlation study. We first investigated the frequency of *IDH1* mutations in our cohort. The mutation rate differed according to WHO grade as follows: 10 of 20 (50.00%) with Grade II, 8 of 30 (26.67%) with Grade III, and 1 of 12 (8.33%) with Grade IV (Table 3). The distribution of *IDH1* mutations among Grade II to IV samples was significantly different based on two-sided Fisher's exact test (*p* = 0.043, Table 3). Our data thus confirmed that the *IDH1* mutation more frequently occurred in low-grade gliomas (Grade II and III) than in high-grade gliomas (Grade IV), in accordance with previous reports.

**Table 3:** Distribution of *IDH1* mutations in glioma patients based on sequencing results

WHO Grade	Wild-type	Mutant	Total	Positive Rate	Significance
II	10	10	20	50.00%	**0.043**
III	22	8	30	26.67%	
IV	11	1	12	8.33%	
Total	43	19	62	30.65%	

**Figure 4:**
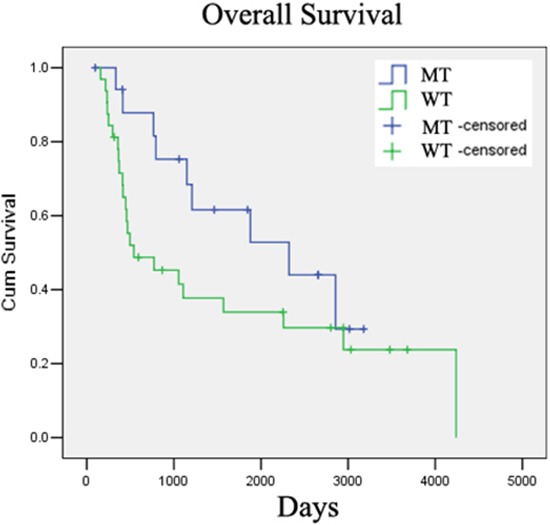
IDH1 mutation is a positive prognostic marker for low-grade glioma patients *IDH1(R132H)* mutation correlated with better overall survival in low-grade glioma patients in our cohort.

It has been reported that the *IDH* mutation is a positive prognostic biomarker of low-grade gliomas (Grade II and III) [19–21]. We next studied the correlation between *IDH1* and the prognosis of low-grade glioma in our cohort (20 with Grade II and 30 with Grade III, 50 total). At a median follow-up of 57.6 months (range from 17.2–139.40 months), the 5-year survival rates were 66.67% and 37.50%, for patients with or without *IDH1* mutation, respectively (*p* = 0.048, Table 4). We used two Kaplan-Meier survival analysis methods, the Log-rank and Breslow tests, to evaluate the role of the *IDH1* mutation in the prognosis of glioma patients (Grade II and III). The overall survival rate of patients with wild-type and mutant *IDH1* was 28.13% and 50.00% respectively, which was significantly different by Breslow analysis (*p* = 0.038), but not by Log-rank analysis (*p* = 0.132, Table 4, Figure 4). The reason for the discrepancy on overall survival between two analysis methods may be the limited size of our cohort (50 in total). Furthermore, we detected only the mutations on *IDH1* but not on *IDH2*, which may have led to the inconsistency in the correlation analysis. We therefore confirmed that the *IDH1* mutation is a positive prognosis marker in low-grade glioma patients.

**Table 4:** Survival differences between wild-type and mutant *IDH1* glioma patients (Grade II and III)

	Survival Rate (%)		Significance
Five-year	Wild-type	37.50	0.048(Chi-Square)
	Mutant	66.67	
Overall	Wild-type	28.13	0.132 (Log-Rank) 0.038 (Breslow)
	Mutant	50.00	

## DISCUSSION

Publications in the 2015 *New England Journal of Medicine and Nature Genetics* confirmed the positive role of mutant *IDH* in glioma evolution and prognosis. The TCGA research group performed genome-wide analyses in 293 low-grade gliomas [20]. They then classified low-grade gliomas into following types: type I, with both *IDH* mutant and *1p19q* codeletion; type II, with *IDH* mutant only; and type III, with wild-type *IDH*. Natsume et al. [21] also found that low-grade gliomas were composed of three subtypes, similar to TCGA's classification. These two studies yielded consistent results and revealed that *IDH1* mutation correlated with both the specific mutation profiles and distinctive clinical behaviors better than traditional histology-based grouping. Eckel-Passow et al. [19] scored tumors according to the *IDH* mutation, mutations in the *TERT* (telomerase reverse transcriptase) promoter, and the codeletion of *1p19q* in 1087 gliomas and 11590 controls. The molecular groups were independently associated with overall survival among patients with Grade II and III but not among patients with Grade IV gliomas. Among patients with Grade II and III gliomas, survival was best in the *IDH* and *TERT* mutation group. These molecular classifications reveal that the metabolic reprogramming caused by mutant IDH may occur early in glioma development. Accordingly, the critical role of *IDH* mutations in molecular diagnosis, determination of therapeutic strategy, and prediction of prognosis was established.

Digital PCR is an end-point method that quantifies nucleic acids without standard curves and is independent of reaction efficiency. The principle of digital PCR, which is the same as that of qRT-PCR, is specific amplification of a nucleic acid target. The distinctive characteristic of digital PCR depends on the partitioning and the subsequent statistical analysis of PCR product distribution across the partitions. The ddPCR technique used in our study employed advanced microfluidics technology to achieve partitioning on a large scale, generating nearly 20,000 highly uniform nanoliter-sized droplets per sample, which yielded a high volume of data points and enabled quantitative measurements at a new level of accuracy. As a simple and reliable technique, ddPCR has been applied in many fields, such as cancer biomarker detection, infectious diseases, genomic alterations, and gene expression.

In obesity, ddPCR was employed together with whole-genome sequencing to identify the copy number variations of the amylase gene locus [22]. Albano et al. detected the PML-RARA transcript in 76 newly diagnosed acute promyelocytic leukemia (APL) cases using ddPCR [23]. In DNA methylation detection, ddPCR showed greater precision, accuracy, and technical simplicity than qRT-PCR [24]. Early detection of the ESR1 mutation with ddPCR in breast cancer biopsies allowed for the cessation of ineffective endocrine therapies and the initiation of other treatments without the need for tissue biopsy [25]. Morerover, the ddPCR approach could be used for patient follow-up with tumor-specific circulating nucleic acids if the tumor mass is unavailable. Data from ddPCR revealed comparable absolute miRNA concentrations and consistent trends of dysregulation in breast cancer patients compared with controls [26]. The BRAF mutant in the circulating DNA of melanoma plasma was successfully detected by ddPCR, which fell with treatment response and rose with detectable disease progression. [27].

In our study, we compared the characteristics of ddPCR and qRT-PCR and found that ddPCR was much more sensitive than qRT-PCR. Another study compared ddPCR and qRT-PCR by detecting the copy number of demethylated CpG promoter sites of the CD3Z gene. The statistical analysis showed that linear concordance was stronger for ddPCR than qRT-PCR, and the absolute values obtained by ddPCR were closer to flow cytometry results [24]. With regard to the false-positive outcome in our study, we suggest confirming the threshold setting strictly, although the manufacturer claimed that the method is not threshold-dependent. Another question is which one is more sensitive, ddPCR or direct sequencing. Guttery DS et al. validated next-generation sequence data with ddPCR in their study and found that ddPCR worked better than next-generation sequencing [25]. Those rare mutations (as low as 0.1%) could be identified by ddPCR but may be considered noisy in the case of direct sequencing. Been the standard method for mutation detection, Sanger direct sequencing takes longer processing time, which hinders its clinical application. It is possible that ddPCR may replace direct sequencing in the near future.

We successfully applied ddPCR to detect the frequent mutation of *IDH1* in glioma patient tissue samples in the current study. Compared with Sanger direct sequencing and qRT-PCR, ddPCR is a simple and sensitive method with which to detect site mutations and could be widely applied in cancer to detect, for example, mutations, copy number variations, and circulating nucleic acids. The application of ddPCR in cancer would improve cancer diagnostic efficiency and facilitate treatment response monitoring in the future.

## MATERIALS AND METHODS

### Patient tissue samples

Glioma specimens were obtained from the Department of Neurosurgery/neuro-oncology of Sun Yat-sen University Cancer Center (SYSUCC) from 2001 to 2007 with written informed consent (*n* = 62). Patients were diagnosed and classified by the Department of Pathology at SYSUCC following the World Health Organization (WHO) guidelines, 20 with Grade II, 30 with Grade III, and 12 with Grade IV glioma. There were 40 females and 22 males, with ages ranging from 2 to 74 years (average, 40.48 years). This investigation was approved by the SYSUCC institutional review board and was conducted in accordance with the ethical standards of the Declaration of Helsinki and international and national guidelines.

### Follow-up

The follow-up records of all patients included in this study was lastly updated in October 30, 2014. After the completion of therapy, patients were observed at 3-month intervals during the first 3 years and at 6-month intervals thereafter. Five-year survival and overall survival were defined as the time from surgery to the five-year follow-up (1825 days) and the date of death or the last date of contact if patients were still alive, respectively.

### Droplet digital PCR

ddPCR was performed at the Department of Health Technology and Informatics of The Hong Kong Polytechnic University. The reaction mixture for ddPCR contained 66 μg of patient tissue DNA, 900 nmol/L forward and reverse primers, 250 nmol/L FAM-labeled WT probe, 250 nmol/L VIC-labeled R132H probe, and 10 μl of 2 × ddPCR™ Supermix for Probes (BioRad Laboratories, Pleasanton, CA, USA). Distilled water was added to achieve a final volume of 20 μl. The primer sequences for *IDH1* were the following: Forward, 5′-CGG TCT TCA GAG AAG CCA TT-3′, Reverse, 5′- ATT CTT ATC TTT TGG TAT CTA CAC C-3′. The fluorescent Taqman MGB probes (FAM-labeled-WT-5′- ATC ATA GGT CgT CAT GCT TAT -3′ and VIC-labeled R132H mutant -5′- ATC ATA GGT CaT CAT GCT TAT -3′) were synthesized by Life technology (Grand Island, NY, USA). The reaction mixture was then partitioned into nanoliter-sized droplets using QX200 Droplet Generator™ (BioRad Laboratories), in which the target and background DNA was randomly distributed into the droplets. Then, the droplets were transferred to a 96-well plate for PCR reaction in a thermal cycler (2720, Life technologies, Grand Island, NY, USA). The PCR program was initiated and held at 95°C for 10 min, followed by 40 cycles at 94°C for 30 sec, 56°C for 1 min, and 98°C for 10 min. The PCR product from each well was then subjected to the QX200 Droplet Reader (BioRad Technologies), which analyzed the fluorescence of each droplet individually using a two-color detection system. Custom software (QuantaSoft; BioRad Technologies) was used to define the graphical areas or “gates” associated with each allele type and to count the number of droplets within each gate.

### Quantitative real-time PCR

Quantitative real-time PCR (qRT-PCR) was performed with ABI PRISM 7500 Real Time PCR System (Applied Biosystems, Bedford, MA, USA) with Platinum^®^ Quantitative PCR SuperMix-UDG (Invitrogen, Carlsbad, CA, USA) with the same primer pair and probes as those used in ddPCR. The thermal cycling conditions were the following: hold for 5 min at 95°C, followed by 40 cycles at 95°C for 1 min, 56°C for 45 sec, and 72°C for 2 min, and ending with a hold at 72°C for 7 min. The amplification curves of the wild-type and mutant were studied to determine the *IDH1* mutation status. Data were analyzed with the ABI Prism Sequence Detection Software (Applied Biosystems). The threshold cycle (Ct, the number of cycles at which the fluorescence exceeds the threshold) was recorded for further analysis [16].

### Sanger sequencing

The experiment was performed at the Department of Molecular Diagnosis at SYSUCC. The first round of PCR consisted of 125 ng of genomic DNA, 12 μM forward and reverse primer (Invitrogen, Carlsbad, CA, USA), 25 μl of 2 × PCR Master mix (Tiangen Biotech, Beijing, China). Distilled water were added to achieve a 50-μl final volume. The Department of Molecular Diagnosis at SYSUCC provided the primers. PCR amplification cycling consisted of an initial denaturation step at 94°C for 5 min; 35 cycles at 94°C for 30 sec, 58°C for 30 sec, and 72°C for 30 sec; and a final extension at 72°C for 5 min. The reactions were performed using a Thermal Cycler (2720, Life Technologies). The PCR products were separated on an agarose gel (2%, Life Technologies) and purified according to the instructions of the QIAquich Gel Extraction Kit (Qiagen, CA, USA). Products were then subjected to the second round of amplification using the BigDye Terminator v3.1 Sequencing Kit (Applied Biosystems). The amplification program was started at 96°C for 1 min, followed by 25 cycles at 96°C for 10 sec, 50°C for 5 sec, 60°C for 4 min, and a hold at 4°C. After a purification step using a BigDye Xterminator Purification Kit (Applied Biosystems), both forward and reverse sequences were determined using an ABI prism 3500xL DNA analyzer (Applied Biosystems), and data were collected using the ABI Prism 310 Data Collection Software.

### Serial dilution of DNA samples

To evaluate the difference in the detection sensitivity of ddPCR and qRT-PCR, we diluted the samples and used both methods. A sample with a strong mutant *IDH1(R132H)* signal was chosen and diluted with double-distilled water at the following ratios: 1:8, 1:16, 1:32, 1:64, 1:128, 1:256, and 1:512. At these ratios, the total amount of genomic DNA per reaction was 8.25, 4.125, 2.063, 1.031, 0.516, 0.258, and 0.129 ng, respectively. The diluted samples were then subjected to ddPCR and qRT-PCR analysis.

### Statistical analysis

The overall survival of glioma patients (Grade II and III) with wild-type or mutant *IDH1* was investigated by Kaplan-Meier survival analysis, including the Log-rank (Mantel Cox) and Breslow tests. Five-year survival rate of patients (Grade II and III) were analyzed by Chi-square test between those with or without *IDH1* mutation. The differences in the *IDH1* mutation rate in gliomas (Grade II to IV) were analyzed by the Fisher's exact test. All *p* values were two sided, and *p* < 0.05 was considered statistically significant. Statistical analysis was performed without SPSS 19.0 (SPSS, Inc., Chicago, IL).

## SUPPLEMENTARY MATERIALS TABLE


