# Supplementary material for: IDH1 mutation detection by droplet digital PCR in glioma
Source: Oncotarget. 2015 Oct 14;6(37):39651–60. doi: 10.18632/oncotarget.5630 (PMC4741852; doi:10.18632/oncotarget.5630)
Supplement: Supplementary file 1 [file oncotarget-06-39651-s001.pdf]

SUPPLEMENTARY TABLE

No.	WHO grade	Gender	Age	KPS	Ending	Survival time (Days)	IDH1 mutation
1	2	1	55	90	1	540	WT
2	2	1	33	90	1	1055	WT
3	2	1	43	90	1	1208	R132H
4	2	2	46	100	1	1879	R132H
5	2	2	25	10	1	2258	WT
6	2	1	17	60	0	2801	WT
7	2	2	43	70	0	2659	R132H
8	2	1	31	80	0	2654	R132H
9	2	2	56	90	0	2945	WT
10	2	1	35	60	0	1845	R132H
11	2	1	56	90	1	2856	R132H
12	2	1	37	90	1	2946	WT
13	2	2	44	100	0	3179	R132H
14	2	2	56	90	0	408	R132H
15	2	2	43	50	0	1463	R132H
16	2	1	4	100	0	3679	WT
17	2	2	2	90	0	3480	WT
18	2	2	35	70	0	315	WT
19	2	1	30	100	0	1060	R132H
20	2	2	30	80	1	4240	WT
21	3	1	35	80	1	158	WT
22	3	1	53	100	1	215	WT
23	3	1	54	70	1	232	WT
24	3	1	65	70	1	235	WT
25	3	1	33	80	1	250	WT
26	3	1	50	70	1	297	WT
27	3	1	31	80	1	333	R132H
28	3	1	58	70	1	357	WT
29	3	1	38	90	1	364	WT
30	3	1	32	60	1	371	WT
31	3	1	10	90	1	410	WT
32	3	2	43	60	1	412	R132H
33	3	1	9	80	1	415	WT
34	3	1	57	100	1	447	WT

(*Continued*)

No.	WHO grade	Gender	Age	KPS	Ending	Survival time (Days)	IDH1 mutation
35	3	2	59	50	1	457	WT
36	3	1	54	80	1	467	WT
37	3	1	65	80	1	495	WT
38	3	1	70	90	1	766	R132H
39	3	1	60	90	1	771	WT
40	3	2	47	80	1	793	R132H
41	3	2	37	90	1	1107	WT
42	3	1	30	90	1	1149	R132H
43	3	1	57	60	1	1569	WT
44	3	2	44	90	1	2322	R132H
45	3	1	39	70	0	97	R132H
46	3	1	39	80	0	592	WT
47	3	2	49	60	0	3015	R132H
48	3	2	33	80	0	3032	WT
49	3	1	19	90	0	2254	WT
50	3	1	17	90	0	866	WT
51	4	1	56	90	1	69	WT
52	4	2	41	60	1	299	WT
53	4	2	74	80	1	301	WT
54	4	2	26	90	1	313	WT
55	4	1	52	90	1	363	WT
56	4	1	44	100	1	397	WT
57	4	1	29	90	1	523	R132H
58	4	1	21	80	1	830	WT
59	4	1	8	50	1	1485	WT
60	4	2	51	80	0	2205	WT
61	4	1	44	60	0	1614	WT
62	4	1	56	50	0	2584	WT

Gender:1, female, 2,male
Ending: 1, dead, 0, alive.
